# Preliminary Evidence on Intra-Articular Autologous Conditioned Serum (ACS) in Temporomandibular Joint Disorders (TMDs): A Systematic Review with a Focus on Mechanisms and Potential Application in Clinical Practice

**DOI:** 10.3390/ijms26188798

**Published:** 2025-09-10

**Authors:** Marcin Pasternak, Maciej Chęciński, Kamila Chęcińska, Natalia Turosz, Izabella Chyży, Bartosz Kosiński, Klaudia Kwiatkowska, Kalina Romańczyk, Amelia Hoppe, Maciej Sikora

**Affiliations:** 1 Division of Clinical Pharmacology, Department of Pharmacology, Jagiellonian University Medical College, 16 Grzegórzecka Street, 31-531 Cracow, Poland; marcin.pasternak@uj.edu.pl; 2 National Medical Institute of the Ministry of the Interior and Administration, Wołoska 137 Str., 02-507 Warsaw, Poland; kamila.checinska@pimmswia.gov.pl (K.C.); natalia.turosz@pimmswia.gov.pl (N.T.); sikora-maciej@wp.pl (M.S.); 3 Department of Maxillofacial Surgery, Hospital of the Ministry of the Interior and Administration, Wojska Polskiego 51, 25-375 Kielce, Poland; 4 Department of Anesthesiology and Intensive Care, Leszek Giec Upper-Silesian Medical Center, Medical University of Silesia in Katowice, Ziołowa 45–47, 40-635 Katowice, Poland; ichyzy@gcm.pl; 5 Faculty of Architecture, Civil Engineering and Applied Arts, Academy of Silesia, Rolna 43, 40-555 Katowice, Poland; bartosz.kosinski@akademiaslaska.pl; 6 Department of Oral Surgery, Preventive Medicine Center, Komorowskiego 12, 30-106 Cracow, Poland; klaudia011998@wp.pl (K.K.); kalina.romanczyk@wp.pl (K.R.); amelia.a.hoppe@gmail.com (A.H.); 7 Department of Biochemistry and Medical Chemistry, Pomeranian Medical University, Powstańców Wielkopolskich 72, 70-111 Szczecin, Poland

**Keywords:** temporomandibular joint, temporomandibular disorders, intra-articular injections, autologous conditioned serum

## Abstract

Intra-articular injections form a substantial element of the temporomandibular joint disorder (TMD) therapy. Given the role played by IL-1β in pathology, the use of autologous conditioned serum (ACS) is well-founded. Despite years of effective use in different locations, data regarding the intra-articular administration of ACS in TMD is scarce, and the strategy itself is not routinely applied. This study aims to provide preliminary evidence on the therapeutic efficacy of administering intra-articular ACS in treating TMD. Patients with TMD who received intra-articular ACS were included. More invasive co-interventions, such as arthroscopy, were excluded. Final searches were conducted on 17 June 2025, using five databases (ACM, BASE, DOAJ, PubMed, and SciELO). Risk of bias was evaluated using the RoB 2 tool. The results were tabulated. Only one study met the inclusion criteria. When compared to dextrose prolotherapy in internal TMD, ACS therapy resulted in greater improvement in mouth opening, pain, and joint-sound reduction. The small sample size, head-to-head design, and limited blinding suggest a highly cautious interpretation of the findings. ACS is a promising, but still experimental, therapeutic strategy addressing critical mechanisms in TMD. However, the currently available data is insufficient to confirm the effectiveness and safety of such an approach, and further high-quality studies are needed. This study received no funding. PROSPERO registration number: CRD420251069310.

## 1. Introduction

The management of temporomandibular joint (TMJ) dysfunctions remains challenging, as current therapeutic strategies often fail to address the underlying pathology directly. Autologous conditioned serum (ACS) has recently been investigated as a potential treatment option in temporomandibular disorders (TMDs) [1]. The risk of systemic side effects, in turn, is a concern related to the pharmacotherapy, like NSAIDs or corticosteroids use. The risk increases with the chronic administration of high doses, and such a regimen usually is needed in the pathology discussed [2]. Local hyaluronic acid intra-articular injections form a promising alternative. Randomised trials, however, are inconclusive regarding the efficacy of the method and the time of pain relief obtained, which lowers initial enthusiasm [1]. The lack of a solid base for employed therapies forms an important question in the dental practice and maxillofacial surgery, also, as the TMJ—the only double joint in the human body—is not free from the pathology of this type. With an estimated incidence in the world population equal to 34%, TMDs are, in fact, one of the most common oral and facial pain conditions [3].

Irrespectively of the affected joint location, osteoarthritis and other closely related disease entities show a remarkable pattern of mechanisms with a highly orchestrated interplay between the cytokines and other pro-destructive agents. The local, pro-inflammatory cytokines release initiates the process of the demolition of the hyaline cartilage and its matrix. Immediately after, the following, even greater intracellular cytokine release results in further cartilage destruction. The damage is at this point more severe than in the beginning, as, concurrently, the natural matrix turnover is hampered [4]. Within a short time, the balance between the protective and destructive elements is disrupted. The positive feedback loop of a vicious cycle of pathology intensifies the production and release of the noxious factors.

**Figure 1:**
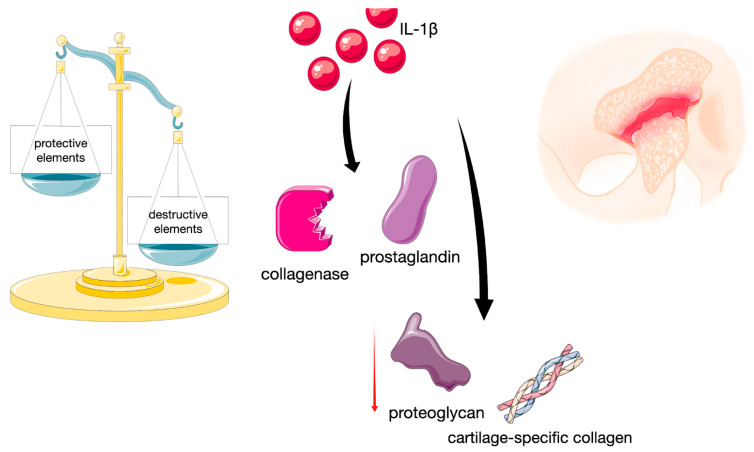
Diagram illustrating process leading to joint damage as a result of an imbalance between protective and destructive elements. Independently composed of original drawing and images provided by Servier Medical Art (https://smart.servier.com/), licensed under CC BY 4.0 (https://creativecommons.org/licenses/by/4.0/).

Interleukin-1β (IL-1β) appears to be a factor of vital importance in the development of the pathology described (Figure 1) [5]. IL-1β is indispensable in collagenase and prostaglandins production, and simultaneously limits the synthesis of proteoglycans and cartilage-specific collagen, thus escalating inflammation and strengthening the process of destruction (Figure 1) [6].

**Figure 2:**
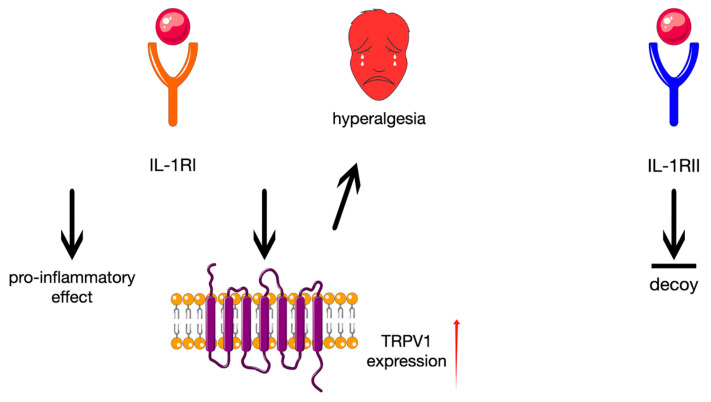
Schematic representation of the response induced by IL-1RI and IL-1RII. The figure is composed of images provided by Servier Medical Art (https://smart.servier.com/), licensed under CC BY 4.0 (https://creativecommons.org/licenses/by/4.0/).

The cytokine stimulates its appropriate type I and II type of receptors (IL-1RI and IL-1RII, respectively) [7]. Activation of the first is mainly responsible for pro-inflammatory effects, while the other is a decoy receptor and does not play any important role in the intracellular signalisation [8]. Additionally, the IL-1RI stimulation increases transient receptor potential vanilloid 1 (TRPV1) expression in nociceptors, which is critical for the subsequent hyperalgesia induction (Figure 2) [5].

Scientific and clinical data confirms that high levels of the cytokine’s competitive receptors antagonist—IL-1ra—limit the pernicious work of IL-1β [4]. The main mechanism responsible is the cytokine’s receptors inhibition. Since concentrations of IL-1ra in the affected tissues are usually too low to stop the disease progression, an increase in IL-1ra levels is a desirable target for therapy [4]. Already, over twenty years ago, a gene therapy enhancing the IL-1ra synthesis was proven to be effective in an animal model of osteoarthritis [9]. This aim may be fulfilled, however, in other, more predictable ways, including ACS therapy [4].

ACS is still not a strategy, that is employed in everyday dental practice, although it addresses mechanisms important also in the pathology of TMD. For this reason, we decided to perform a systematic search on studies assessing the attempts of practical use of the method, preferably high-quality trials, which would allow for comparison with other approaches using intra-articular injections in TMD.

The assessment of the therapeutic efficacy of intra-articular ACS administration in afflictions of TMJ is the main objective of this systematic review. We hope to clarify the question of ACS implementation in TMD, by synthesizing the evidence available in the literature, ideally gaining evidence-based insights. Such findings could be useful in clinical practice and would pave the way for decisive future research, which is currently lacking, though much needed, in this field of therapy.

## 2. Materials and Methods

This article reports the results of a PROSPERO-preregistered (CRD420251069310), PRISMA 2020-compliant systematic review of the therapeutic efficacy of intra-articular conditioned serum for the treatment of TMD. Eligibility criteria were determined using the PICOTS method, and searches for medical articles were performed using different engines using the same query. Clinical trials with all levels of evidence were included in the review, but the meta-analysis was based exclusively on randomized controlled trials. Each source article was assessed for risk of bias using a tool dedicated to the given article type. Detailed methodological aspects are presented in the following subsections.

### 2.1. Eligibility Criteria

The review included studies in which the population consisted of patients with diagnosed TMDs, in the absence of a joint prosthesis on the assessed side. The intervention had to consist of ACS without concomitant invasive procedures such as arthroscopy. For comparative purposes, quantitative analyses included control groups using placebo or another potentially therapeutic intra-articular substance, provided that no other surgical procedure was performed.

The analysis focused on clinical variables such as articular pain, mandibular range of motion, and health-related quality of life. Studies that did not present results in a form that allowed for quantitative analysis were excluded from the meta-analysis, although they could be included qualitatively. There were no time limits on publication dates. Only articles published in English were included, and access to the full text of the publication was required.

The full list of inclusion and exclusion criteria adopted is presented in Table 1.

### 2.2. Information Sources and Search Strategy

The strategy included (1) database searches conducted using the same query for all engines, (2) gray literature searches using the Google Scholar engine (with “allintitle:” filter), (3) reference review, and (4) adding to the pool of records items known to the team from research experience in the analyzed topic.

Based on the above inclusion and exclusion criteria, a query for search engines was developed. The query was refined by conducting preliminary searches using the following engines: (1) Bielefeld Academic Search Engine (BASE) and (2) National Institutes of Health: National Library of Medicine: National Center for Biotechnology Information (PubMed). The final database search engines used were (1) ACM Guide to Computing Literature, (2) Bielefeld Academic Search Engine (BASE), (3) Directory of Open Access Journals (DOAJ), (4) National Library of Medicine PubMed, and (5) Scientific Electronic Library Online (SciELO).

Gradual refinement of the query with the aim of maximum inclusiveness resulted in its final form: “(temporomandibular OR tmj OR tmd) AND (“conditioned serum” OR “autologous serum” OR “anti-inflammatory serum” OR “anti-inflammatory serum” OR antiinflammatory serum OR acs OR “interleukin-1 receptor antagonist” OR “IL-1Ra” OR orthokine OR regenokine OR anakinra OR kineret) AND (“intra-articular” OR intraarticular OR “intra-articularly” OR intraarticularly OR “intra-cavitary” OR intracavitary) AND (injection OR injections OR injected OR injecting OR administration OR administrations OR administered OR administering OR deposition OR depositions OR deposited OR depositing)”. Final database searches were conducted on 17 June 2025 and gray literature searches ended on 31 July 2025 (M.C., K.K.).

### 2.3. Selection and Data Processing

The study selection process was conducted in two stages and was independently performed by two review authors (M.C., K.C.). In the first stage, titles and abstracts of publications identified from the searches in medical articles, conference proceedings, and dissertations were analyzed. In the second stage, a full-text review of eligible reports (M.C., M.P.) was performed to confirm their compliance with previously established inclusion and exclusion criteria (Table 1). Rayyan (version 2025, Rayyan Systems Inc., Doha, Qatar) was used to support the selection process, allowing for independent and blinded assessment of the decision to include or exclude. Any discrepancies in the assessment were resolved in the first stage in favor of the record (promotion to full-text review) and in the second stage by discussion and voting with the participation of a third reviewer (M.S.).

Data from eligible studies were also extracted independently by two authors (K.C., K.R.). A standardized form developed in the Google Sheets spreadsheet, which is part of the Google Workspace package (version 2025, Google LLC, Mountain View, CA, USA), was used for extraction. The form included general information about the publication (authors, year of publication), participant characteristics (including age, sex, TMD diagnosis), details of the intervention and comparison (type, number and method of injection), measured outcomes (arthralgia, range of mandibular mobility, quality of life) and the moments of their assessment. In case of disagreement between extractors, the data were re-analyzed until a consensus was reached. In cases of ambiguity regarding results or methodology, an attempt was made to extract data based solely on the available content of the publication.

### 2.4. Risk of Bias Assessment

The risk of bias in individual studies was assessed independently by two members of the review team (M.C., A.H.) using appropriate tools depending on the type of study. For randomized clinical trials, the Cochrane RoB 2 (Risk of Bias 2) tool was be used, while for non-randomized studies, the ROBINS-I (Risk Of Bias In Non-randomized Studies—of Interventions) tool was intended to be used. As specified in the protocol, uncontrolled studies were not intended to be assessed for risk of bias and were not included in the analyses. Accordingly, they were not considered as evidence for the purpose of drawing quantitative conclusions from the review, but were taken into account in exploring the level of interest in ACS, identifying adverse events, and related contextual aspects. In the event of discrepancies in assessments, the decision was be made by consensus or with the involvement of a third reviewer.

### 2.5. Effect Measures and Synthesis Methods

Continuous outcome measures related to key clinical outcomes such as arthralgia, mandibular range of motion, and health-related quality of life were included. Variables were transformed to 0–10 cm VAS, mm, percentages, respectively. Data are presented as mean differences (MDs), each with 95% confidence intervals.

The decision to include individual studies in the syntheses was made based on their characteristics tabulated, including type of intervention, comparator groups, and reported endpoints. Studies that did not meet analytical criteria (e.g., ineligible study type, missing quantitative data) were intended to be excluded from the individual syntheses but included in the review for method development and identification of complications.

Incomplete data were planned to be processed according to Cochrane guidelines—for example, missing standard deviations estimated using standard errors, confidence intervals, or *p* values. Where data were only presented graphically, they were to be extracted using the most accurate digital readout possible.

The results were presented with Google Workspace package (version 2025, Google LLC, Mountain View, CA, USA). For this purpose, scatter plots with regression model fitting (selection of the best-fitting model) were intended to be presented, illustrating changes in individual results over time, as well as forest plots—for key time points common to a larger number of studies.

## 3. Results

### 3.1. Study Selection

**Figure 3:**
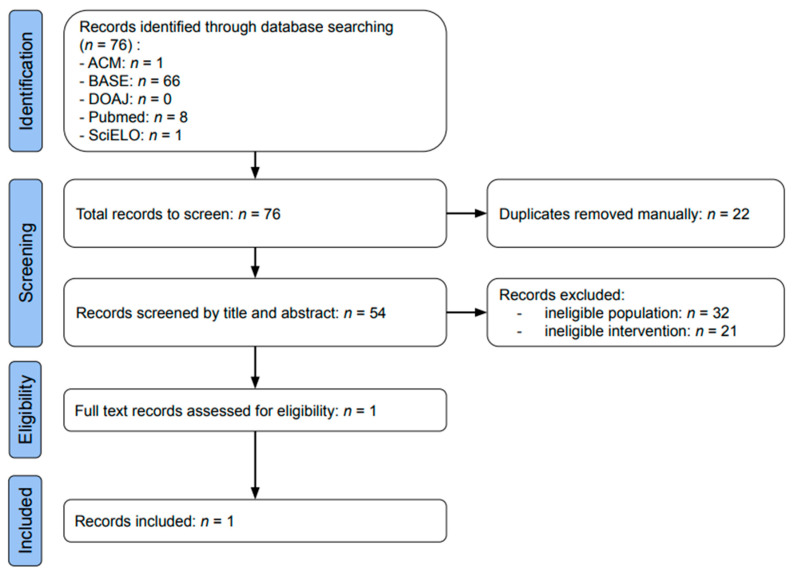
Flow diagram of the study identification, screening, eligibility, and inclusion process.

The selection process is presented in Figure 3. Data searches using the ACM, BASE, DOAJ, PubMed, and SciELO databases identified 1, 66, 0, 8, and 1 result, respectively (Table A1). During manual deduplication prior to screening, 22 articles were eliminated. The remaining 54 results were assessed based on titles and abstracts, with 32 excluded due to ineligible population and 21 due to ineligible intervention. Only one article was included in the review: “Evaluation of the efficacy of autologous conditioned serum compared with dextrose prolotherapy in internal temporomandibular joint disorders—a pilot study”, C. Ravikumar, 2024 [1].

### 3.2. Study Characteristics

The above article describes a pilot study involving a treatment group (intra-articular administration of ACS) and a control group (dextrose prolotherapy). Both groups consisted of 12 healthy adults (aged 18–50 years) diagnosed with unilateral temporomandibular joint (TMJ) dysfunction and experiencing persistent symptoms for more than 6 months.

Patients were evaluated for pain intensity using the Visual Analog Scale (VAS, 0–10), mouth opening range (mm), mandibular deviation (mm), and the presence or absence of joint sounds in the preauricular area. These parameters were assessed in both groups after 2 weeks, 1 month, and 2 months.

### 3.3. Risk of Bias in Studies

In the study by C. Ravikumar et al., 2024, it was shown that only the assessors were blinded during the research process [1]. This raises some concerns regarding patients’ perception of TMJ pain. Other evaluated factors (jaw deviation, mouth opening and joint sounds) were either objectively measurable (in millimeters) or assessed by blinded examiners, and the risk of bias for these outcomes is considered low. Table A2 presents the risk of bias assessment.

### 3.4. Results of Individual Studies

The only study meeting the inclusion criteria was conducted by Ravikumar et al., who compared intra-articular ACS with dextrose prolotherapy in patients with internal derangement of the TMJ. Full details of the methodology and outcomes, including TMJ pain, mouth opening, deviation, and joint sounds, are available in the original publication [1].

## 4. Discussion

The outcomes of the only trial that met the criteria of this systematic review indicate that ACS may be regarded as a valuable tool in TMD treatment, particularly in the most popular intracapsular form of TMD—TMJ internal derangement (TMJ disc displacement). Addressing etiopathogenesis in the most direct way, the method has a distinct advantage over currently applied therapies using intraarticular administration. The results regarding pain alleviation and improvement in mouth opening and joint sounds are encouraging. The conduction of much larger studies, preferably comparing ACS with placebo and all standard therapeutical approaches is still needed, similarly to the inclusion of patients with other types of TMD. The afflictions of the TMJ occupy a significant place among the orthopedic disorders. Multifarious nature, varying and complex etiology and the existence of various sub-diagnoses, such as myofascial pain and TMJ inflammation, are among their chief characteristics. Simultaneously, in TMD as in other kindred afflictions, the same pattern of mechanisms behind the pathology is present [5]. As in other locations, IL-1β plays a vital role both in expanding and maintaining the inflammation [5]. It is also one of the first cytokines released by macrophages penetrating the synovium in several TMJ issues, starting with a pathologic overload of the joints. Furthermore, in synovial fluid from patients with TMD, IL-1β is the most prevalent cytokine to be found [5,10]. The beneficial processes, such as a growth factor secretion, protecting the intra-articular tissues from similar noxious stimuli, are restricted in TMD and IL-1ra levels decrease [4,11,12].

As a rule, conservative measures are recommended as the initial approach in the management of TMD. Only when non-surgical methods fail, should more invasive, surgical techniques, even involving arthroplasty, be considered. An interesting intervention, helpful in TMD and bridging the gap between conservative and surgical procedures, is arthrocentesis—the lavage of the affected joint, without exhibiting its interior [13]. Sterile needles and irrigant are used, and inflammatory mediators’ removal results in the pain reduction. Through the means of hydraulic pressure of irrigation, intra-articular adhesions are eliminated, and mandible mobility is thus increased [13]. Intra-articular administration of solutions other than irrigative agents can be employed in TMD management [14]. ACS appears as an interesting alternative there. Except for direct action in the tissues affected, the strategy addresses the mechanisms behind the pathology in the broadest way, among the options available [1,4,15].

**Figure 4:**
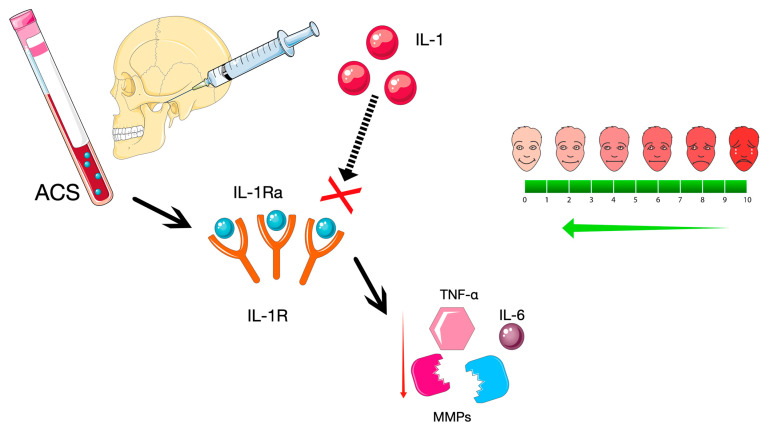
Expected effect of ACS injected into the temporomandibular joint in TMD. The figure is composed of images provided by Servier Medical Art (https://smart.servier.com/), licensed under CC BY 4.0 (https://creativecommons.org/licenses/by/4.0/).

ACS, in short, can be described as an autologous blood product enriched in the interleukin-1 receptor antagonist (IL-1Ra). The beneficial cytokine is one of the most important, but not the only, ACS active ingredient (Figure 4) [4].

**Figure 5:**
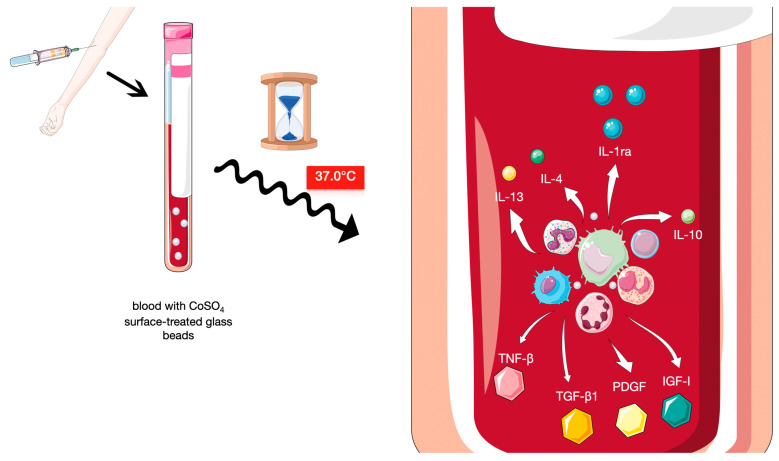
Schematic representation of the ACS preparation process and the subsequent cellular response, including the release of anti-inflammatory and anti-destructive agents. The figure is composed of images provided by Servier Medical Art (https://smart.servier.com/), licensed under CC BY 4.0 (https://creativecommons.org/licenses/by/4.0/).

The method was suggested in the mid 1990s. From that time on, endeavours for its implementation had been made in several experimental and clinical settings, and the strategy itself is constantly refined [15]. The base of the technique is formed by the exposure of leukocytes from a patient’s blood to pyrogen-free surfaces of medical-grade glass beads. Meijer et al. were the first to demonstrate that after this kind of incubation a rapid synthesis of several factors occurs [16]. Among the anti-inflammatory and anti-destructive properties of agents accumulated in the liquid phase of blood prepared, there is IL-1ra, followed by other beneficial cytokines, like IL-4, IL-10 and IL-13, with a number of growth factors, like insulin-like growth factor-1 (IGF-1), platelet-derived growth factor (PDGF), and transforming growth factor-β1 (TGF-β1) [15]. Intriguingly, 24 h exposure to the CrSO_4_ surface-treated glass beads increases IL-1ra concentration even up to 140-fold, without similar impact on pro-destructive agents. The levels of IL-1β and tumour necrosis factor beta (TNF-β), two well-known pro-inflammatory elements important in this kind of pathology, at the same time, were virtually unaltered (Figure 5) [17].

After the centrifugation and extraction, thus-prepared samples can be either stored or injected into affected tissues. The length of blood sample incubation described in the literature varied in research conducted, with time values like 3 h, 6 h, 6–9 h and even 24 h reported. The temperature of incubation in all studies remains the same, and close to the body temperature, with a value equal to 37.0 °C (98.6 °F) [1,17,18,19]. A notable advantage of ACS is the fact that it requires only one injection [1]. Other strategies employing intra-articular administration on average require more than one injection, and such a course prevails in clinical practice. In trial conditions, one can find isolated reports on singular injections of PRP or I-PRF [20].

As it was already said before, ACS therapy is not the only method using intra-articular injection [1]. The solutions used are endowed with anti-inflammatory, lubricative, and regenerative properties, while the mode of injection itself allows for exact, direct administration in the affected region [21]. The strategies other than ACS, and, at the same time, most popular in clinical practice, include the intra-articular administration of corticosteroids, dextrose prolotherapy (DP), hyaluronic acid (HA), or platelet-rich plasma (PRP) [1]. Unlike ACS therapy, in these methods, more than a single injection is required. A varying number of appointments is needed to obtain acceptable effects lasting for a differing amount of time [22]. Each of these approaches has its advantages and limitations in TMD management.

Corticosteroids form a group of natural hormone analogues and derivatives with a well-known anti-inflammatory action. These agents increase expression of annexin-1 (other name: lipocortin-1), a potent inhibitor of phospholipase A2—the enzyme essential for inflammatory cytokines synthesis—impairing in this way the arachidonic acid release and restricting further synthesis of its various derivatives Corticosteroids also affect the regulation of apoptosis in thymocytes—T cell progenitors [23]. Another important mechanism is the blocking of proinflammatory cytokines transcription, especially IL-1β, IL-6 and TNF-α [22]. Corticosteroids employed in intra-articular injections vary in terms of duration of exerted action. Hydrocortisone, methylprednisolone and triamcinolone are most appropriate for this manner of administration. These agents are water-insoluble and form microcrystalline particles, the level of the drug is lower, and its steady release lasts longer. Dexamethasone and betamethasone sodium phosphate, in turn, are water-soluble non-particulates; their effect is rapid, but it is a shorter lasting one [23]. In addition to the potential side effects already mentioned, the sole way of administration of these drugs is related to unwanted outcomes. The chondrotoxicity and subsequent cartilage destruction is more probable with repeated intra-articular injections [22]. Obviously, that risk severely restricts the chronic implementation of corticosteroids in intra-articular therapy [1]. It was observed also, that in temporomandibular osteoarthritis management, the beneficial effects were limited to pain alleviation, with little or no effect on other symptoms, like mouth opening. Furthermore, these outcomes were not long-lasting ones [24,25]. The ineffectiveness of intra-articular corticosteroid administration compared with placebo in recent studies was significantly marked in studies with groups with a higher number (>70%) of female patients, but this issue requires further clarification [25].

With the first attempts made already in 1937, DP is a strategy that, among the listed, has been administered for the longest time in TMJ pathologies [26]. The active ingredients of the solution injected are dextrose and local anaesthetic, each component mixed in a syringe shortly before administration [1,27]. The effects resemble physiological sequelae of the healing process. The initial inflammatory response right after injection is particularly important. Immune system cells, granulocytes, monocytes, and macrophages are attracted, and the release of growth factors occurs (chiefly EGF, IGF, PDGF, TGFβ, basic fibroblast growth factor—BFGF—and connective tissue growth factor—CTGF). The recent literature emphasises the multifactorial nature of therapeutic mechanisms induced, highlighting, at the same time, the role of direct sensorineural effects [28]. Analgesic effect results from nerve hyperpolarisation, due to neuronal potassium channels opening, with a subsequent decrease in signal transmission. Dextrose solution, most probably, is blocking transient receptor potential vanilloid type 1 (TRPV 1), an aim of several strategies used for combating the neuropathic pain [28]. Furthermore, dextrose (d-glucose) itself is an eminent nutrient for cartilage and the precursor for glycosaminoglycan, glycoprotein, and glycolipid synthesis, facilitating regeneration [29]. The data concerning the efficacy in TMD management, despite longtime use of DP, is still inconclusive. It is generally agreed that the method relieves pain, but the time of the effect reported in studies varies [28,30]. Disparities in outcomes were observed in different pathologies treated. Improvement in mouth opening, for example, was observed in patients with TMJ internal derangement, while the same effect was scarce or even non-existent according to the broader systematic review of research on DP use in various types of TMD [28,30]. In the case of patients with internal derangement of the TMJ, DP brought relief from pain, though it failed to induce long-lasting effects. It was particularly evident when the strategy was compared with ACS. The latter, in addition, improved symptoms other than pain [1]. The DP adverse effects reported include a burning sensation after the injection and transitory paralysis of the facial nerve (temporal branch) [28].

The lubricating properties of hyaluronic acid are an unquestionable advantage. Unfortunately, however, the degree of lubrication obtained after intra-articular administration in clinical practice may not be sufficient. It was observed that within the affected TMJ, the agent fails to minimize friction within the joint in a satisfying manner [22]. Furthermore, hyaluronic acid exerts no noticeable influence on the biomechanical aspect of intra-articular pathology. The agent does not participate in dynamic changes in pro-destructive and anti-inflammatory factors and does not affect the levels of cytokines or growth factors [1]. The relatively high cost of therapy is a considerable limiting factor [31].

PRP is a therapeutic strategy that, among those listed, seems to be most akin to ACS, also employing a patient’s own blood sample. Due to their autologous origin, both methods are relatively safe. While in the case of ACS the sample is incubated with glass beads and the blood and serum components are together centrifuged afterwards, the older method uses processed plasma, employing centrifugation without incubation to isolate the serum phase and the concentrate with thrombocytes within [1]. Importantly, anticoagulant use is required before the centrifugation [32]. The differences in PRP preparation methods and technical parameters used may affect, in a significant manner, properties of the obtained product. For example, a change in the speed of centrifugation affects the composition of the sample, as the greater velocity employed results in higher concentrations of growth factors [33]. In terms of PRP, among the other counterparts listed, this seems to be most closely on a par with ACS, regarding addressing the biomechanical aspects of the treated pathologies. Administered into affected TMJ, it stimulates the process of release of several important growth factors. They include not only platelet-derived growth factor (PDGF), but also epidermal growth factor (EGF), fibroblast growth factor (FGF), insulin-like growth factor (IGF), transforming growth factor (TGF), and vascular endothelial growth factor (VEGF) [1]. Surprisingly, the role of the latter is at least ambiguous. Its receptors are present on the surface of the osteoarthritic joint and absent on normal adult chondrocytes [34]. VEGF in osteoarthritis acts as an autocrine stimulator, mediating chiefly destructive processes [34]. The question to what extent these actions are counterbalanced by other factors remains open, and, similarly, whether VEGF may limit beneficial processes induced by those agents [32]. Therefore, the moderate increase inVEGF levels or no change in its concentration, as observed in some animal studies, may form an additional advantage of ACS over the older method [35]. Table 2 provides a closer look at the active components present in high concentrations in samples prepared with both methods. Particular attention is given to those that are important for TMD intra-articular therapies [1,35].

Encouraging outcomes of PRP therapy are marked in the management of pathologies affecting joints which are different from TMJ. In the case of TMD, the effects seem to be less certain. The specific characteristics of the only human double-joint and its pathologies are the most probable explanation. Knee joints, responding well to the intra-articular PRP administration in comparison with TMJ, are characterized by a higher amount of synovial tissue, which is one of the main targets of the therapy [32]. The pain intensity decrease is certainly an outcome appreciated by the patients. Unfortunately, the effect obtained, similarly to improvement in acoustic symptoms or mouth opening, is not a long-lasting one, as the therapeutic efficacy of this strategy diminishes over time [32].

Interestingly, in clinical practice, the last two approaches (i.e., PRP and HA) could be combined. Asadpour and colleagues found that, following arthrocentesis, in ailment control of symptomatic temporomandibular joint osteoarthritis (TMJ-OA), the combined strategy was more effective than each of the two methods employed alone [36]. The conclusions from a recent systematic review suggest the superiority of the combined approach in TMD therapy, although the difference was statistically insignificant, probably due to the low number of patients in the studies assessed [37]. However, more research is needed to answer the question of whether combined PRP and HA therapy can be considered better than ACS or the last technique is superior to the combination, as is the case with each of the two approaches used alone [15,25].

Several limitations of this review should be acknowledged. Despite searching five databases using a refined query, only one eligible study was identified, which limits the comprehensiveness of the synthesis and makes the interpretation more challenging. Our focus on ACS inevitably narrowed the search scope; a broader search window, including other intra-articular biological products such as platelet-rich plasma, might have yielded more eligible studies and provided a wider context. In the included study, only the assessors were blinded, which may increase the risk of bias. Nevertheless, considering the limited availability of high-quality research in this topic, this work addresses a significant gap in the existing literature.

## 5. Conclusions

The available evidence on intra-articular ACS for TMD is extremely limited, as only one small pilot study met the inclusion criteria. This is insufficient to establish its clinical efficacy or safety. While ACS remains a theoretically promising approach that targets relevant pathological mechanisms and may stimulate regenerative processes, current data do not allow for definitive conclusions. Further high-quality, well-designed studies are essential to clarify its potential role in TMD management.

## Figures and Tables

**Table 1:** Eligibility criteria.

	Inclusion Criteria	Exclusion Criteria
Population	TMD patients	Patients with a TMJ prosthesis on a given side
Intervention	Intra-TMJ administration of ACS	More invasive co-intervention, e.g., arthroscopy
Comparison (applicable to specific analyses only)	Placebo or other intra-articular substance	More invasive comparison, e.g., including arthroscopy
Outcomes (applicable to specific analyses only)	Articular pain, mandibular mobility, or health-related quality of life	Results that cannot be quantified
Timeframe	Any	Not applicable
Study types	Any clinical studies for review inclusion, randomized controlled trials for specific analyses	Not applicable
Publication form	English-language scientific articles	Reports without full text access

**Table 2:** The active components of ACS and PRP important for TMD intra-articular therapy present in higher concentrations than in unprocessed blood.

ACS	PRP
IL-1RA, IL-4, IL-10, IL-13, EGF, FGF, TGF-β, PDGF, IGF, VEGF *	PDGF, TGF, VEGF, IGF, FGF, and EGF

* The concentrations of the last factor, unlike other ones, in comparison to unprocessed blood, may be increased slightly or remain unchanged.

## Data Availability

The protocol of this systematic review is available in the Prospective Register of Systematic Reviews (PROSPERO) under number CRD420251069310. All collected data are included in the content of this article.

## Abbreviations

The following abbreviations are used in this manuscript:

ACS	Autologous Conditioned Serum
BFGF	Basic fibroblast growth factor
CTGF	Connective tissue growth factor
DP	Dextrose prolotherapy
EGF	Epidermal growth factor
FGF	Fibroblast growth factor
HA	Hyaluronic acid
IGF	Insulin-like growth factor
IGF-1	Insulin-like growth factor-1
IL-1Ra	Interleukin-1 Receptor Antagonist
MMPs	Matrix metalloproteinases
PDGF	Platelet-derived growth factor
PRP	Platelet-rich plasma
ROBINS-I	Risk Of Bias In Non-randomized Studies—of Interventions
TGF	Transforming growth factor
TGF-β1	Transforming growth factor-β1
TMD	Temporomandibular disorders
TMJ	Temporomandibular Joint
TMJ-OA	Temporomandibular joint osteoarthritis
TNF-β	Tumour necrosis factor beta
TRPV 1	Transient receptor potential vanilloid type 1
VAS	Visual Analog Scale
VEGF	Vascular endothelial growth factor

## Appendix A

**Table A1:** The total number of records identified and database coverage on the day of the search.

Search Engine	Searched Entries	Identified Items
ACM	3,910,450	1
BASE	424,706,948	66
DOAJ	11,212,063	0
PubMed	Over 38,000,000	8
SciELO	14,407,809	1

**Table A2:** Risk of bias assessment.

	D1	D2	D3	D4	D5	Overall
TMJ pain (VAS)	Low	Some concerns	Low	Some concerns	Low	Some concerns
Mouth opening (mm)	Low	Low	Low	Low	Low	Low
Deviation (mm)	Low	Low	Low	Low	Low	Low
Joint sounds	Low	Low	Low	Low	Low	Low

D1—Risk of bias arising from the randomization process; D2—Risk of bias due to deviations from intended interventions; D3—Risk of bias due to missing outcome data; D4—Risk of bias in measurement of the outcome; D5—Risk of bias in selection of the reported result.
